# Earlywood and Latewood Stable Carbon and Oxygen Isotope Variations in Two Pine Species in Southwestern China during the Recent Decades

**DOI:** 10.3389/fpls.2016.02050

**Published:** 2017-01-10

**Authors:** Pei-Li Fu, Jussi Grießinger, Aster Gebrekirstos, Ze-Xin Fan, Achim Bräuning

**Affiliations:** ^1^Key Laboratory of Tropical Forest Ecology, Xishuangbanna Tropical Botanical Garden, Chinese Academy of SciencesMenglun, China; ^2^Institute of Geography, University of Erlangen-NürnbergErlangen, Germany; ^3^World Agroforestry CentreNairobi, Kenya

**Keywords:** stable carbon isotope, stable oxygen isotopes, intrinsic water use efficiency, subtropical pine species, Asian summer monsoon, intra-annual resolution

## Abstract

Stable isotopes in wood cellulose of tree rings provide a high-resolution record of environmental conditions, yet intra-annual analysis of carbon and oxygen isotopes and their associations with physiological responses to seasonal environmental changes are still lacking. We analyzed tree-ring stable carbon (δ^13^C) and oxygen (δ^18^O) isotope variations in the earlywood (EW) and latewood (LW) of pines from a secondary forest (*Pinus kesiya*) and from a natural forest (*Pinus armandii*) in southwestern China. There was no significant difference between δ^13^C_EW_ and δ^13^C_LW_ in *P. kesiya*, while δ^13^C_EW_ was significantly higher than δ^13^C_LW_ in *P. armandii*. For both *P. kesiya* and *P. armandii*, δ^13^C_EW_ was highly correlated with previous year’s δ^13^C_LW_, indicating a strong carbon carry-over effect for both pines. The intrinsic water use efficiency (iWUE) in the earlywood of *P. armandii* was slightly higher than that of *P. kesiya*, and iWUE of both pine species showed an increasing trend, but at a considerably higher rate in *P. kesiya*. Respective δ^13^C_EW_ and δ^13^C_LW_ series were not correlated between the two pine species and could be influenced by local environmental factors. δ^13^C_EW_ of *P. kesiya* was positively correlated with July to September monthly mean temperature (MMT), whereas δ^13^C_EW_ of *P. armandii* was positively correlated with February to May MMT. Respective δ^18^O_EW_ and δ^18^O_LW_ in *P. kesiya* were positively correlated with those in *P. armandii*, indicating a strong common climatic forcing in δ^18^O for both pine species. δ^18^O_EW_ of both pine species was negatively correlated with May relative humidity and δ^18^O_EW_ in *P. armandii* was negatively correlated with May precipitation, whereas δ^18^O_LW_ in both pine species was negatively correlated with precipitation during autumn months, showing a high potential for climate reconstruction. Our results reveal slightly higher iWUE in natural forest pine species than in secondary forest pine species, and separating earlywood and latewood of for δ^18^O analyses could provide seasonally distinct climate signals in southwestern China.

## Introduction

Due to the intense human impact, including large scale forest harvesting and resultant land use changes, the area of natural forests in tropical and subtropical China is remarkably decreasing in favor of secondary forest or plantation (Fang et al., 2001; Piao et al., 2009). The transformation of land use and vegetation types may have strong impacts on the local climate and the regional hydrological cycle (Li et al., 2009). Hence, it is of urgent importance to understand the responses of different vegetation types to climate factors and environmental change, especially in tropical and subtropical areas in southwestern China that are exposed to extreme summer monsoon precipitation events which may trigger devastating flood events.

Stable carbon and oxygen isotopes from tree rings (δ^13^C, δ^18^O) are frequently used in environmental research, as they provide a continuous, annually resolved record of environmental conditions and show stronger correlations between tree individuals and environmental variables than radial tree growth (McCarroll and Loader, 2004). Tree-ring δ^13^C is controlled by the balance between stomatal conductance and photosynthetic rate (Farquhar et al., 1980; Leavitt and Long, 1988). Thus δ^13^C is frequently correlated with air humidity or precipitation in dry environments (Gebrekirstos et al., 2009, 2011; Kress et al., 2009; Brienen et al., 2011), whereas it is associated with irradiance factors and growing season temperature in humid environments (McCarroll and Loader, 2004). δ^13^C has been widely used to calculate intrinsic water use efficiency (iWUE) and to estimate differences in water use among different plants (McCarroll and Loader, 2004; Gebrekirstos et al., 2011; Gessler et al., 2014). Recent studies reported an increase of iWUE with elevated CO_2_ in sub-tropical and tropical regions (Brienen et al., 2011; Xu Y. et al., 2014).

Tree-ring δ^18^O is mainly controlled by the source water δ^18^O, leaf water exchange and xylem/phloem water exchange and thus reflects variations in hydroclimate (McCarroll and Loader, 2004). In high mountain regions of southwestern China, δ^18^O is a good indicator for monsoon moisture (An et al., 2012) or cloudiness (Liu et al., 2014). δ^18^O in *Fokienia hodginsii* is a promising proxy of drought variability in Laos (Xu et al., 2011). δ^18^O in teak (*Tectona grandis*) is positively correlated with annual precipitation in western and central India, but a negative correlations with annual precipitation was found in southern India (Managave et al., 2011). The combined analysis of δ^13^C and δ^18^O is a promising way to investigate tree physiological responses to environmental changes (McCarroll and Loader, 2004). However, there are only few studies that investigated the tree-ring carbon and oxygen isotope variations in subtropical and tropical areas in southwestern China (An et al., 2012; Xu Y. et al., 2014).

The earlywood in tree rings is produced during spring and early summer, while the latewood is produced in late summer and autumn, thus earlywood and latewood may potentially record climatic signals in different seasons (Fritts, 1976/1987). In addition, wood formation may be influenced by carbohydrates synthesized in the previous growing season and remobilized to form earlywood in the following spring (Kagawa et al., 2006). Such a carbon carry-over effect was proven in the tropical conifer *Podocarpus falcatus* from the Ethiopian highlands (Krepkowski et al., 2013), whereas Kress et al. (2009) showed that Scots pine did not rely on stored carbon reserves from previous years at tree-line sites in European mountain regions. Thus, the contribution of the carry-over effect to wood formation might differ between species and regions. In regions influenced by the Asian summer monsoon climate, An et al. (2012) found that earlywood δ^18^O was affected by early monsoon season temperature and relative humidity (RH), whereas latewood δ^18^O was correlated with late monsoon precipitation and RH. Therefore, separate analyses in tree ring earlywood and latewood stable isotopes may shed light on the physiological responses of tree species to seasonal environmental change.

The secondary forest formed by dominant stands of *Pinus kesiya* Royle ex Gord var. *langbianensis* is an important type of natural forest replacement and have a wide distribution across southern Yunnan, southwestern China (Li et al., 2015). These secondary forests were established after the destruction the natural evergreen *Lithocarpus* forest in 1970s (Young and Wang, 1989). *Pinus armandii* Franchet is a regional natural forest species, which is able to survive in higher elevations than *P. kesiya*. The two pine species share a very similar wood anatomy and form clearly distinguishable growth ring boundaries, which is not very common among tree species of this subtropical ecosystem. Furthermore, the annual growth rates of the two pine species are rather high, and show a clear color distinction between earlywood and latewood, enabling us to carry out intra-annual stable isotope analysis. We analyzed the tree-ring stable carbon (δ^13^C) and oxygen (δ^18^O) isotope variations in the earlywood and latewood of the two pine species. Our aims were (1) to study whether the earlywood and latewood of the two pine species differed in their δ^13^C and δ^18^O and if δ^13^C in earlywood are influenced by a carry-over effect of carbohydrates assimilated in the previous growing season; (2) to investigate if there exist differences in iWUE of pine species from the secondary forest and the natural forest and their response to elevated CO_2_; (3) to detect the seasonal climatic signals that control tree ring δ^13^C and δ^18^O in earlywood and latewood of the studied species. We hypothesized that (1) natural forest pine would have higher iWUE than the secondary forest pine; (2) tree ring δ^18^O in earlywood and latewood of both pine species might capture seasonal moisture signals (precipitation, relative humidity), that earlywood recorded early growing season moisture signal, while latewood recorded autumn moisture signal.

## Materials and Methods

### Study Site and Climate

**FIGURE 1:**
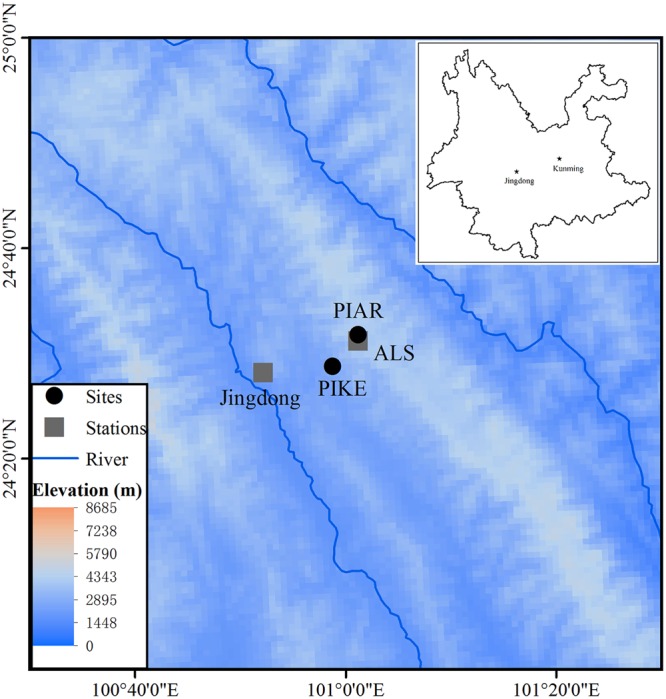
**Locations of sampling sites of *P. kesiya* (PIKE) and *P. armandii* (PIAR) and meteorological stations in the Ailaoshan Station for Subtropical Forest Ecosystem Studies (ALS) and Jingdong County within Yunnan Province, southwest China (in lay map)**.

This study was carried out in a subtropical forest ecosystem of the Ailao Mountains, Yunnan Province, southwestern China. The Ailao Mountains form the major climatic border between regions influenced by the southwestern Asian summer monsoon and East Asian summer monsoon (Young and Wang, 1989), and harbors remnants of natural evergreen subtropical forest in higher elevations above 2000 m a.s.l. Local climate data are available from the Ailaoshan Station for Subtropical Forest Ecosystem Studies (ALS) (1982–2012, 24°31′ N, 101°01′ E, 2480 m a.s.l), and a longer data record covering the period 1956–2012 from a weather station in Jingdong County, located 60 km far from ALS in a valley (24°28′ N, 100° 52′ E, 1162.3 m a.s.l.) (**Figure 1**).

**FIGURE 2:**
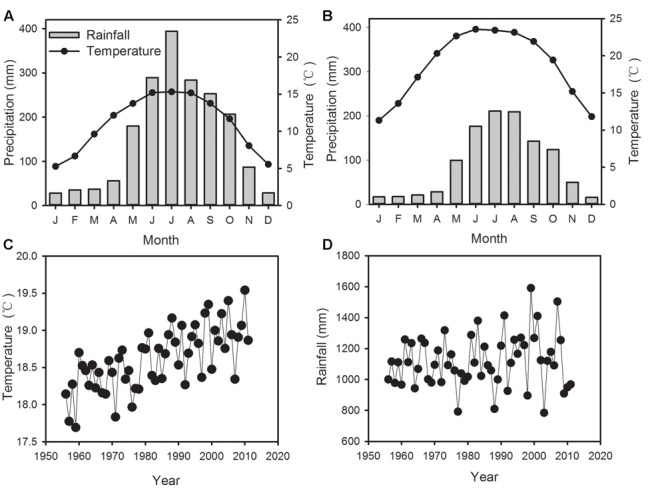
**Monthly mean temperature and precipitation in Ailaoshan Station for Subtropical Forest Ecosystem Studies (ALS) (A)** and in Jingdong County **(B)**, time series of annual mean temperature **(C)** and precipitation **(D)** in Jingdong County from 1956–2012.

The mean annual precipitation at ALS is 1880 mm, of which more than 86% falls during the rainy season from May to October. The mean annual temperature is 11°C, with a minimum of 5.3°C in January and a maximum of 15.3°C in July (**Figure 2A**). In January, temperature may drop below 0°C. The investigated forest is a subtropical cloud forest with heavy fog during June to August and November to December. Annual mean precipitation at Jingdong County amounts 1118 mm and mean annual temperature is 18.6°C (**Figure 2B**). Although differing in absolute values, the temporal variations of temperature and precipitation between Jingdong County and ALS are synchronous, therefore the data of both stations are highly correlated (Supplementary Table S1, **Figure 2**). However, RH during the late summer and autumn months shows poor correlation between the two stations (Supplementary Table S1), since ALS is mainly situated in a dense cloud cover during summer months while Jinglong is located in a river valley. Hence, we used temperature and precipitation data from Jingdong with longer record but RH data from ALS for further correlation analysis.

### Study Species and Tree-Ring Sampling

*Pinus kesiya* var. *langbianensis* is a geographic variant of *P. kesiya*, and *P. kesiya* has a wide distribution in SE Asia, occurring in Myanmar, India, Laos, Vietnam, Thailand, Philippines, and some African countries (Armitage and Burley, 1980). For short, we used ‘*P. kesiya*’ instead of ‘*P. kesiya* var. *langbianensis*’ in the following parts of this paper. *P. kesiya* is the dominant tree species in the secondary forest of Ailao Mountains and is associated by *Schima noronhae* at elevations between 700 and 2000 m a.s.l. *Pinus armandii* can be found in mountain areas and river basins at elevations ranging from 1000 to 3300 m a.s.l. of southwestern China. In our study sites, this species is distributed in remnants of the natural Lithocarpus forests above 2000 m a.s.l. According to the dendrometer data, stem growth of both *P. kesiya* and *P. armandii* started in April and ended in October (Supplementary Figure S2).

We collected two increment cores for each tree, and 40 cores (20 trees) of *P. kesiya* from a secondary pine forest (24°29′ N, 100°59′ E, 1980 m a.s.l.), and 36 cores (18 trees) of *P. armandii* from a natural forest close to ALS (24°32′ N, 100°1′ E, 2495 m a.s.l.). The stem density in the tree layer of the *P. kesiya* is lower (1,032 stems ha^-1^ and canopy cover less than 50%) than those of the natural *Lithocarpus* forest (21,400 stems ha^-1^ and canopy cover more than 95%) (Young and Wang, 1989). The sampling site of *P. kesiya* is located on a north-facing slope with an inclination of *ca.* 10°, and the soil type is Nitisol. The local farmers continuously harvest the resin of *P. kesiya* when the tree diameter at breast height (DBH) exceeds 20 cm (Wang et al., 2006). There are no evidences of human managements, such as logging, irrigation and fertilization. The sampling site of *P. armandii* is a north facing slope with an inclination of *ca.* 20°, and the soil type is Luvisol.

Tree-ring widths were measured with traditional technique under the stereomicroscope linked to a LINTAB digital positioning table with a resolution of 0.001 mm (LIBTAB^TM^ 6, Rinntech, Germany). Tree-ring measurements were cross-dated to the calendar year of their formation by growth pattern matching, and statistical tests using the software package TSAP-Win (Rinn, 2003). A visual dating was applied for *P. kesiya*, since the trees are mostly younger than 30 years. Tree ring-width chronology of *P. armandii* spanned from 1906 to 2010, with an average growth rate of 3.34 mm per year (Supplementary Figure S1). The mean sensitivity (0.426) and high inter-series correlation (*r* = 0.66) indicate the reliable quality of the ring-width chronologies.

We selected cores from each of six *P. kesiya* individuals and each of five *P. armandii* individuals for further analysis of δ^13^C and δ^18^O. The average tree ages for selected trees is 32 ± 4 years. for *P. kesiya*, and 45 ± 5 years. for *P. armandii*, respectively. To better disentangle seasonal influences on the δ^13^C and δ^18^O variations in the tree-rings, earlywood (EW) and latewood (LW) of the annuals rings were cut separately with a razor blade, according to their bright and dark color.

### Cellulose Extraction

Alpha-cellulose was extracted by using the Multiple Samples Isolation Systems according to Wieloch et al. (2011) and Qin et al. (2015). Resin, fatty acids, etheric oils, and hemicellulose were extracted with a solution of 5% NaOH for 2 h at 60°C for two times. Then lignin was extracted with 7% NaClO_2_ solution at 60°C, an 8 h × 5 times process was applied in order to achieve an adequate chemical treatment. The remaining hemicelluloses were extracted with 17% NaOH for 2 h at room temperature. A washing procedure with boiled water was interposed for three times across different steps. Finally, samples were treated once with 1% HCl and rinsed with boiling de-ionized water for three times and transferred from the filter funnels into Eppendorf tubes with 1 ml de-ionized water. The samples were then homogenized by using an ultrasound unit (Hielscher Ultrasonics, c.f. Laumer et al., 2009). After freeze drying for 72 h in a lyophilisation unit, the dried α-cellulose samples were processed to the mass spectrometry. δ^13^C and δ^18^O were measured with an Elemental Analyzer coupled to a Delta V Advantage IRMS (Thermo Fisher) while laboratory standards were periodically interposed to test analytical replication. The δ^13^C and δ^18^O values were referred to International Standards (VPDB, VSMOW) and their analytical error lie within typically reported analytical precisions δ^13^C = ±0.15′, δ^18^O = ±0.3′

### Calculation of Intrinsic Water Use Efficiency (iWUE)

The measured carbon isotope values were corrected for anthropogenically induced trends of a rising atmospheric CO_2_ (McCarroll and Loader, 2004). The δ^13^C and CO_2_ concentration (*c*_a_) of ambient air during 1956–2004 were obtained from McCarroll and Loader (2004) and those data for 2005–2012 were provided by Prof. Danny McCarroll (University of Swansea, UK).

According to Farquhar et al. (1982), isotope discrimination (Δ) is defined as follows:

(1) $$\begin{array}{c} \Delta \;=\;(\delta^{13}C_{air}\;-\;\delta^{13}C_{plant})/(1\;+\;\delta^{13}C_{air}/1000) \end{array}$$

Where δ^13^C_plant_ and δ^13^C_air_ refer to the δ^13^C values of the α-cellulose and of atmospheric CO_2_, respectively. Δ was also showed to be closely correlated with *c*_i_/*c*_a_ by Farquhar et al. (1982):

(2) $$\begin{array}{c} \Delta \;=\;a\;+\;(b\;-\;a)(c_{i}/c_{a}) \end{array}$$

Where *a* represents isotopic discrimination occurring during diffusion of CO_2_ from the atmosphere into the intercellular spaces of leaves with *a* = 4.4‰; *b* represents the isotopic fractionation through discrimination that occurs during enzymatic carboxylation with *b* = 27‰. By combining Equations (1) and (2), the intercellular CO_2_ (*c*_i_) can be calculated. iWUE was calculated according to Linares and Camarero (2012):

(3) $$\begin{array}{c} iWUE\;=\;A/g_{s}\;=\;(c_{a}\;-\;c_{i})/1.6\\ \;=c_{a}(b\;-\;\Delta )/1.6(b\;-\;a) \end{array}$$

### Statistical Analysis

The carbon and oxygen isotope series of different individuals were analyzed using the numerical mix method to produce a mean isotope chronology via arithmetic average (Liu et al., 2012). To remove the impact of extreme values of individual series on the mean chorology, the individual carbon and oxygen isotope series were standardized by subtracting the long-term mean and dividing them by their standard deviation. The average δ^13^C and δ^18^O chronology for the six individuals of *P. kesiya* and five individuals of *P. armandii* were used to investigate the correlations between mean δ^13^C and δ^18^O chronologies in earlywood and latewood and climate data. The mean Pearson’s correlation coefficients among carbon and oxygen isotope series of tree individuals within each species (Rbar) were calculated to examine the common climate signal carried by individual series. The expressed population signal (EPS; Wigley et al., 1984) was also calculated to estimate the internal coherence of the individual tree-ring time series. Trees may use carbohydrates assimilated during the previous growing season and stored in wood parenchyma to form earlywood in the following year (carry-over effects). Hence, it is possible that climate not only affects plant growth in the same year, but also in the subsequent year. Thus, a period of 18-months from previous year’s July to current December was used for the calculation of correlation coefficients between monthly means of temperature, precipitation and RH and stable isotope variations to determine the climatic factors influencing stable isotope fractionation, and to test if stable isotope ratios in a tree ring were also influenced by previous year’s climate. Differences between EW and LW of δ^13^C and δ^18^O in the two species were tested for significance by using Independent samples *t*-test. We used the fraction model of Waterhouse et al. (2002) to reconstruct the earlywood and latewood growth period source water δ^18^O during the period 1986–2003, and compared these results with the weighted mean δ^18^O of precipitation at the nearest Global Network of Isotopes in Precipitation (GNIP) station, in Kunming (World Metrological Organization station #5677800; 25°02′ N, 102°43′ E, 1895 m a.s.l.).

## Results

### Characteristics of the Stable Carbon and Oxygen Isotope Chronologies

**FIGURE 3:**
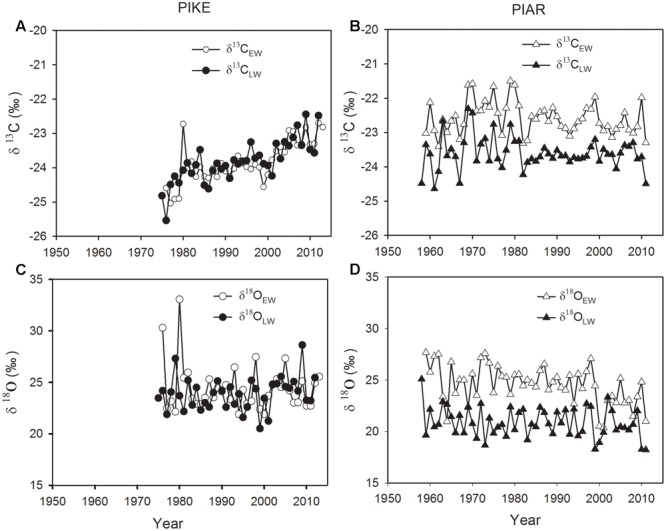
**Carbon isotope variations in earlywood (δ^13^C_EW_, open circle) and latewood (δ^13^C_LW_, closed circle) of secondary forest pine *P. kesiya*** (PIKE, **A**) and in earlywood (δ^13^C_EW_ open triangle) and latewood (δ^13^C_LW_, closed triangle) of *P. armandii* (PIAR, **B**), and oxygen isotope variations in earlywood (δ^18^O_EW_, open circle) and latewood (δ^18^O_LW_, closed circle) of *P. kesiya* **(C)** and in earlywood (δ^18^O_EW_, open triangle) and latewood (δ^18^O_EW_, closed triangle) of forest pine *P. armandii* **(D)**.

Both δ^13^C_EW_ and δ^13^C_LW_ of secondary forest pine *P. kesiya* showed similar mean values (**Figure 3A**, *P* = 0.989, Independent Samples *t*-test), while δ^13^C_EW_ was higher than δ^13^C_LW_ in natural forest pine *P. armandii* (**Table 1**; **Figure 3B**, *P* < 0.001). There was no significant difference between δ^18^O_EW_ and δ^18^O_LW_ in *P. kesiya* (**Figure 3C**, *P* = 0.224), while δ^18^O_EW_ of *P. armandii* was higher than that of δ^18^O_LW_ (**Table 1**; **Figure 3D**, *P* < 0.001). For δ^13^C, the inter-series correlations are higher in earlywood than latewood of both pine species. In contrast, δ^18^O showed higher inter-series correlations in latewood than earlywood for both pine species. There were significant correlations between δ^13^C_EW_ and previous year’s δ^13^C_LW_ in both pine species (**Table 1**). δ^18^O_EW_ was positively correlated with previous year’s δ^18^O_LW_ in *P. armandii*, while there were no significant correlations between δ^18^O_EW_ and previous year’s δ^18^O_LW_ in *P. kesiya* (**Table 1**). δ^13^C_EW_ of *P. kesiya* vs. δ^13^C_EW_ of *P. armandii*, and δ^13^C_LW_ of *P. kesiya* vs. δ^13^C_LW_ of *P. armandii* were not correlated between two pine species, whereas both δ^18^O_EW_ and δ^18^O_LW_ in *P. kesiya* were positively and significantly correlated with those of *P. armandii*, respectively (**Table 2**).

**Table 1:** Statistics of tree-ring carbon and oxygen isotope chronologies for *Pinus kesiya* (1975–2012) and *Pinus armandii* (1958–2011).

	*P. kesiya* (secondary forest)				*P. armandii* (natural forest)			
	δ^13^C_EW_	δ^13^C_LW_	δ^18^O_EW_	δ^18^O_LW_	δ^13^C_EW_	δ^13^C_LW_	δ^18^O _EW_	δ^18^O _LW_
Mean	-23.82	-23.82	24.42	23.88	-22.58	-23.61	24.55	20.87
*SD*	0.61	0.61	2.256	1.58	0.48	0.46	1.83	1.44
Rbar	0.472	0.374	0.305	0.538	0.439	0.273	0.378	0.597
EPS	0.843	0.782	0.725	0.875	0.796	0.652	0.752	0.881
*r* (_EW_y_ to LW_y-1__)	0.607^∗∗^		0.317		0.504^∗∗^		0.374^∗∗^	

*Rbar: mean correlation coefficients among different individual series; EPS: expressed population signal; r (_EW_y_ to LW_y-1__): correlation coefficients between current year’s earlywood chronologies and the previous year’s latewood chronologies. The values with two stars are statistically significant at *P <* 0.01.*

**Table 2:** Correlation coefficients between the mean carbon and oxygen isotope chronologies of secondary forest pine *Pinus kesiya* and natural forest pine *Pinus armandii*.

		*P. kesiya* (secondary forest)				*P. armandii* (natural forest)		
		δ^13^C_EW_	δ^13^C_LW_	δ^18^O_EW_	δ^18^O_LW_	δ^13^C_EW_	δ^13^C_LW_	δ^18^O_EW_
*P. kesiya*	δ^13^C_LW_	0.655^∗∗^						
	δ^18^O_EW_	0.353^∗^	-0.115					
	δ^18^O_LW_	0.429^∗∗^	0.461^∗∗^	0.129				
*P. armandii*	δ^13^C_EW_	-0.171	-0.257	0.111	0.06			
	δ^13^C_LW_	-0.106	-0.080	0.045	0.051	0.767^∗∗^		
	δ^18^O_EW_	-0.351^∗^	-0.312	0.460^∗∗^	-0.099	-0.035	-0.255	
	δ^18^O_LW_	-0.113	0.018	0.228	0.439^∗∗^	0.054	-0.013	0.131

*The values with stars are statistically significant at *^∗^P <* 0.05 and ^∗^*^∗^P <* 0.01.*

Despite of removing the impact of atmospheric δ^13^C on the tree ring δ^13^C, both δ^13^C_EW_ and δ^13^C_LW_ of *P. kesiya* showed an increasing trend (**Figure 3A**). In contrast, both δ^13^C_EW_ and δ^13^C_LW_ of *P. armandii* showed an increasing trend before 1980 and then remain stable after 1980 with reduced inter-annual variability (**Figure 3B**). The δ^18^O_EW_ and δ^18^O_LW_ in *P. kesiya* showed no significant trends (**Figure 3C**), however, δ^18^O_EW_ in *P. armandii* displayed slightly decreasing trends (**Figure 3D**).

### Intrinsic Water Use Efficiency

**FIGURE 4:**
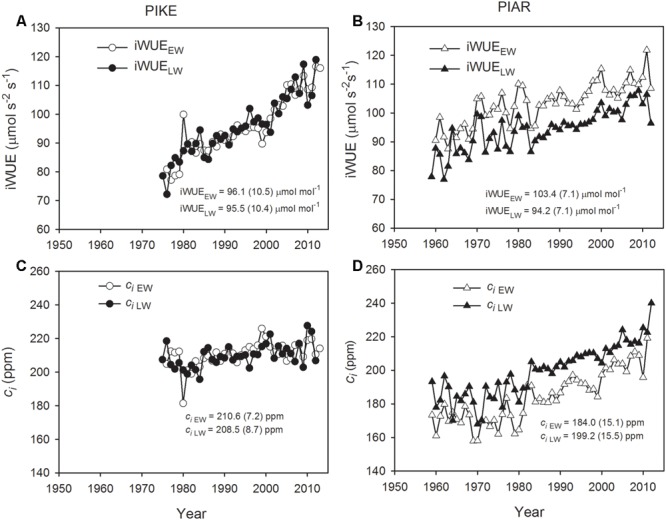
**Intrinsic water use efficiency (iWUE) for earlywood (iWUE_EW_, open circle) and latewood of secondary forest pine *P. kesiya* (PIKE) (iWUE_LW_, closed circle) (A)**, and for earlywood (iWUE_EW_, open triangle) and latewood of natural forest pine *P. armandii* (PIAR) (iWUE_LW_, closed triangle) **(B)**, intercellular CO_2_ concentration (*c_i_*) of earlywood (*c_i_*_EW_, open circle) and latewood of secondary forest pine *P. kesiya* (*c_i_*_LW_, closed circle) **(C)**, and for earlywood (*c_i_*_EW_, open triangle) and latewood of natural forest pine *Pinus armandii* (*c_i_*_LW_, closed triangle) **(D)**. Mean values and standard deviations of iWUE and *c_i_* series are indicated inside the panels.

iWUE in earlywood and latewood of both pine species showed increasing trends from the 1960s to 2010s (**Figures 4A,B**). There was no significant difference in iWUE between earlywood and latewood in *P. kesiya* (*P* = 0.811, **Figure 4A**), while the iWUE in earlywood was consistently higher than that of latewood in *P. armandii* (**Figure 4B**, *P* < 0.001). The earlywood of *P. armandii* had higher iWUE than that of *P. kesiya* (*P* < 0.001). The mean intercellular CO_2_ concentration (*c*_i_) tended to increase with time in both pine species (**Figure 4C**), and *c*_i_ in earlywood was consistently lower than that of latewood in *P. armandii* (**Figure 4D**, *P* < 0.001).

### Correlations of Stable Isotope Chronologies With Climate Factors

**FIGURE 5:**
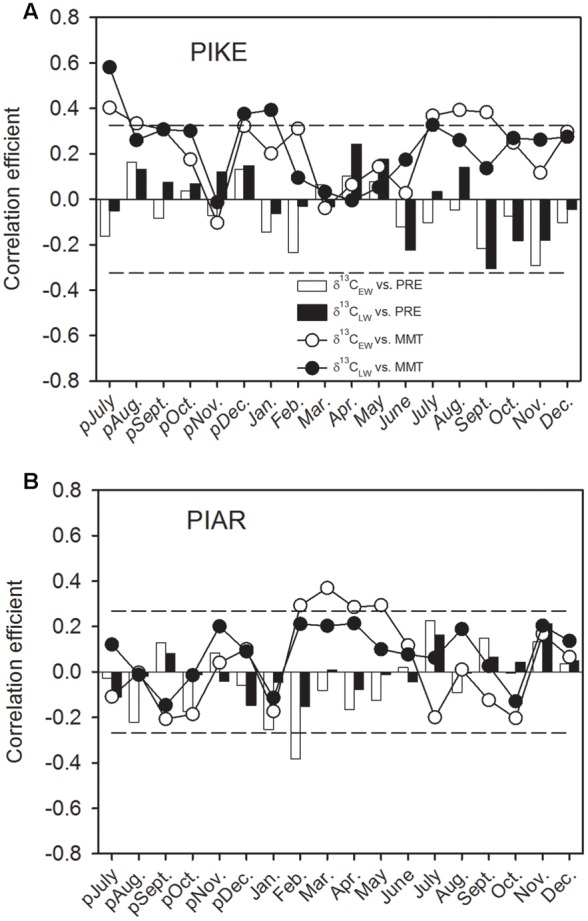
**Correlations of carbon isotope in earlywood (δ^13^C_EW_) and latewood (δ^13^C_LW_) of secondary forest pine *P. kesiya* (PIKE) (1975-2012) (A)**, and natural forest pine *P. armandii* (PIAR) (1958-2011) **(B)** with monthly mean temperature (MMT, open circles for δ^13^C_EW_ and closed circles for δ^13^C_LW_) and monthly precipitation (PRE, open bars for δ^13^C_EW_ and closed bars for δ^13^C_LW_). The dashed lines indicated the 95% confidence intervals.

The δ^13^C_EW_ of *P. kesiya* was positively correlated with monthly mean temperatures (MMT) of the previous year’s July and August, and current July to September (1975–2012), while δ^13^C_LW_ of *P. kesiya* was positively correlated with MMT of previous July and December, and current January and July (**Figure 5A**). We found no significant correlations between δ^13^C_EW_ and δ^13^C_LW_ of *P. kesiya* and precipitation. The δ^13^C_EW_ of *P. armandii* was positively correlated with MMT from February to May (1958–2011), and negatively correlated with monthly precipitation of current February (**Figure 5B**).

**FIGURE 6:**
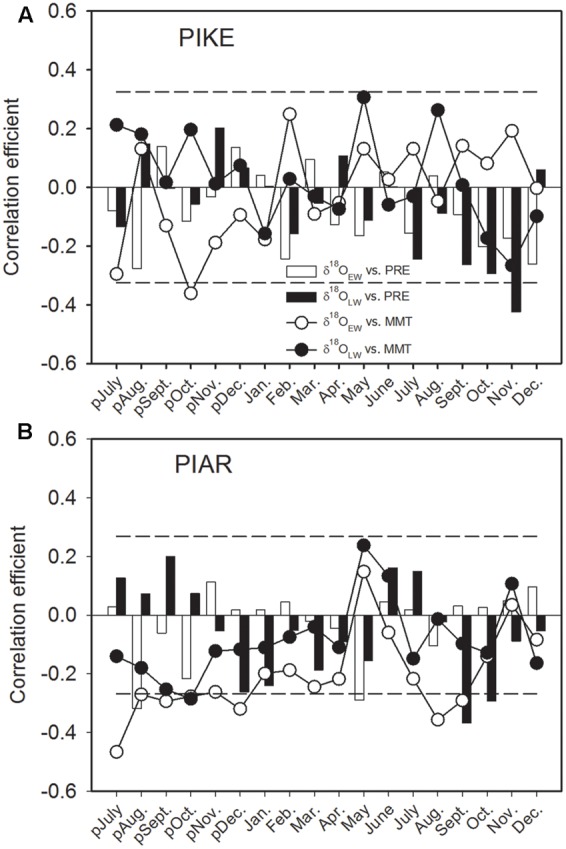
**Correlations of oxygen isotope in earlywood (δ^18^O_EW_) and latewood (δ^18^O_LW_) of secondary forest pine *P. kesiya* (PIKE) (1975-2012) (A)**, and natural forest pine *P. armandii* (PIAR) (1958–2011) **(B)** with monthly mean temperature (MMT, open circles for δ^18^O_EW_ and closed circles for δ^18^O_LW_) and monthly precipitation (PRE, open bars for δ^18^O_EW_ and closed bars for δ^18^O_LW_). The dashed lines indicated the 95% confidence intervals.

The δ^18^O_EW_ of *P. kesiya* showed significantly negative correlation with previous October MMT (1975–2012), while δ^18^O_LW_ of *P. kesiya* correlated negatively with current September, October, and November precipitation (**Figure 6A**). The δ^18^O_EW_ of *P. armandii* was negatively correlated with MMT of previous year’s July to December, and current August and September, and was negatively correlated with previous year’s July and current May precipitation (1958–2011). The δ^18^O_LW_ of *P. armandii* was negatively correlated with current September and October precipitation (**Figure 6B**).

**FIGURE 7:**
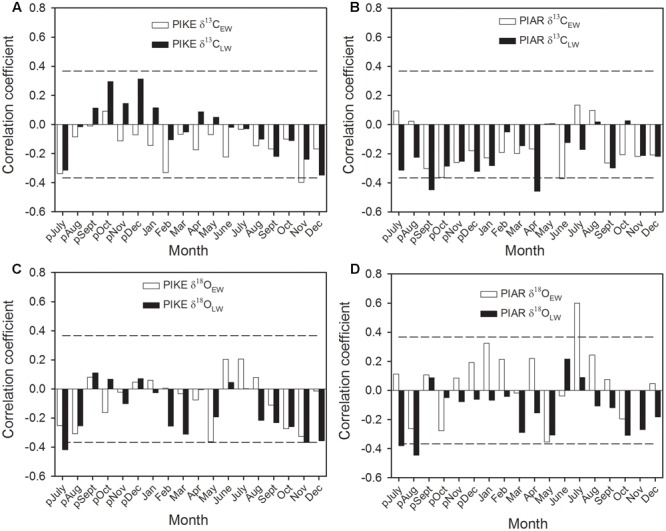
**Correlations of carbon isotope in earlywood (δ^13^C_EW_, open bar) and latewood (δ^13^C_LW_, closed bar) of secondary forest pine *P. kesiya* (PIKE) (A)**, and natural forest pine *P. armandii* (PIAR) **(B)**, oxygen isotope in earlywood (δ^18^O_EW_) and latewood (δ^18^O_LW_) of *P. kesiya* (PIKE) **(C)**, and δ^18^O_EW_ and δ^18^O_LW_ of *P. armandii* **(D)** and relative humidity (RH) from Ailaoshan Station for Subtropical Forest Ecosystem Studies (ALS) (1982–2012). The dashed lines indicated the 95% confidence intervals.

The δ^13^C_EW_ of *P. kesiya* was negatively correlated with November relative humidity (RH) (**Figure 7A**), while while δ^13^C_EW_ of *P. armandii* was negatively correlated with previous year’s October and current June RH, and δ^13^C_LW_ of *P. armandii* was negatively correlated with previous year’s September and current April RH (**Figure 7B**). The δ^18^O_EW_ of both pine species were negatively correlated with May relative humidity (RH), while δ^18^O_EW_ of *P. armandii* was positively with July RH (**Figures 7C,D**). δ^18^O_LW_ of *P. kesiya* was negatively correlated with previous year’s July and November RH, and δ^18^O_LW_ of *P. armandii* was negatively correlated with previous year’s July and August RH (**Figures 7C,D**).

## Discussion

### Intra-annual Variations of Carbon and Oxygen Isotopes

We found that δ^13^C_EW_ are closely related with previous year’s δ^13^C_LW_ in both secondary forest pine *P. kesiya* and natural forest pine *P. armandii*, indicating that there was a significant carbon carry-over effect, which is consistent with Krepkowski et al. (2013). However Kress et al. (2009) showed no carry-over effect for two tree-line conifers due to the limitation of carbohydrates caused by short growing season lengths. Because of the existence of carry over effect in both pine species in the present study, it is suggested to analysis earlywood and latewood carbon isotope separately (Kagawa et al., 2006). The carbon isotopes (δ^13^C) in both earlywood and latewood showed low correlations between *P. kesiya* and *P. armandii* (**Table 2**), indicating that the carbon isotope chronologies reflect mainly species-specific environmental signal.

Since carbon isotopes in tree rings were reported to be possibly affected by age effect, the innermost ca. 30 rings are usually excluded in the carbon isotope study (Schleser et al., 1999). The average age of *P. kesiya* at our site is 30 years., so the carbon isotope series of *P. kesiya* might potentially be affected by age effects. However, the canopy at our study site had a crown density of *ca.* 20% only, so air may ventilate freely between stems. Hence, there is no dense canopy that may possibly cause CO_2_ enrichment of ^13^C depleted air from soil respiration inside the stand, which is the most responsible factor for age effects in tree-ring ^13^C (Schleser et al., 1999; Dorado Liñán et al., 2012). Although we cannot completely exclude any possible age effects on carbon isotope ratios in *P. kesiya*, we consider this ‘age effects’ is negligible in our case.

The seasonal amplitude of δ^18^O is considerably higher in *P. armandii* (3.68‰) than *P. kesiya* (0.56‰) (**Table 1**; **Figures 2C,D**), but these amplitudes are lower than those found from high-altitude fir (*Abies forrestii*) in the nearby Yulong Snow Mountains, southwestern China (*ca.* 8.76‰, An et al., 2012) or from *Fokienia hodginsii* in subtropical lowland China (*ca.* 6.0‰; Xu et al., 2016). The seasonal amplitude of δ^18^O is related to rooting depth, growing season length and sampling resolution, and thus can be species- or site-dependent (Xu C. et al., 2014). Intraspecific δ^18^O_EW_ was uncorrelated with δ^18^O_LW_ for both pine species (**Table 2**), indicating that oxygen isotope variations in the earlywood and latewood represent different climate signals. Moreover, respective δ^18^O_EW_ and δ^18^O_LW_ of natural forest pine are positively correlated with those of secondary forest pine (**Table 2**), pointing to a strong common seasonal climatic forcing imprinted on oxygen isotope. Our results suggest that separating EW and LW δ^18^O may provide seasonally distinct climate information.

### Species-Specific Differences in Water Use Efficiency

Our results showed that pine species from natural forest (*P. armandii*) had a slightly higher iWUE compared to pine species from a secondary forest (*P. kesiya*) (**Figures 4A,B**), indicating that the former could have a more conservative water use than the secondary forest pine species, which is consistent with our first hypothesis. Conservative water use strategy associated with higher iWUE and lower growth rates were also reported from tree species in East Africa (Gebrekirstos et al., 2011). We found that the earlywood of *P. armandii* had higher iWUE than that of latewood (**Figure 4B**), which could be due to the lower water status and reduced stomatal conductance in the early growing season (Qi et al., 2012). However, we found no significant difference between iWUE in earlywood and latewood in *P. kesiya*. One reason may be related to the regular resin harvesting in secondary forest pine which may reduce carbohydrates within the tree in the early growing season (Wu M. et al., 2015).

With the increase of atmospheric CO_2_, the intercellular CO_2_ (*c*_i_) and iWUE in both species have been increasing, which is consistent with previous studies (Xu Y. et al., 2014; Wu G. et al., 2015). The increasing rate of iWUE in secondary forest species (0.82 μmol mol^-1^ per ppm) is twice as high as that of forest pine (0.38 μmol mol^-1^ per ppm), and the increase rate of iWUE of *P. kesiya* is also considerably higher than those found from other coniferous species (Xu Y. et al., 2014).

### Relationships between Intra-annual Stable Isotopes and Climate Factors

We found that inter-series correlations of earlywood δ^13^C were higher than those of latewood δ^13^C for both pine species, whereas δ^18^O showed higher inter-series correlations in latewood than earlywood for both pine species (**Table 2**). Our results indicated that both δ^13^C in earlywood and δ^18^O in latewood of the two pine species carried a stronger common signal that most likely related to climatic factors. Earlywood δ^13^C of both pine species mainly stored a temperature signal (**Figure 5**), while latewood δ^18^O recorded precipitation signal (**Figure 6**).

δ^13^C_EW_ of *P. kesiya* was positively correlated with MMT of previous year’s July and current July to September (1975–2012), however, due to the impact of resin collection on *P. kesiya*, correlations between δ^13^C and climate should be interpreted with care. In contrast, δ^13^C_EW_ of *P. armandii* was positively correlated with MMT from February to May (1958–2011). The spring season is characterized by increasing temperature as well as relatively dry conditions before the onset of the rainy summer monsoon season (**Figure 1**). Stomatal conductance and intercellular CO_2_ concentration would be reduced due to lower soil water status in the spring season, and there would be a weaker carboxylation discrimination against δ^13^C and resulting in positive correlations between δ^13^C_EW_ of *P. armandii* and MMT in the early growing season. In contrast to earlywood, δ^13^C_LW_ of *P. armandii* was not significantly correlated to any climate parameter, which was consistent with the fact that the inter-series correlations of latewood δ^13^C were lower than those from δ^13^C_EW_.

The present study showed that δ^18^O in both *P. armandii* and *P. kesiya* shared common moisture signals, that δ^18^O_EW_ of both pine species was negatively correlated with May RH, and δ^18^O_LW_ of both pine species was negatively correlated with the autumn (September to November) precipitation, which is consistent with our second hypothesis. As can be derived from dendrometer measurements nearby from our study site, cambium growth of *P. kesiya* last until October (Supplementary Figure S2). However, the biomass accumulation and cell wall thickening of latewood cells may even continue until November, which needs to be corroborated by wood anatomical studies. The δ^18^O in the earlywood and latewood of *P. armandii* represent pre-monsoon precipitation and late monsoon precipitation signals, respectively (**Figure 6**). Moreover, we found that the δ^18^O in the earlywood and latewood of both *P. kesiya* and *P. armandii* were negatively with the pre-monsoon (May) RH and autumn RH, respectively (**Figures 7C,D**), which were also convinced by other studies (An et al., 2012; Xu et al., 2016; Zeng et al., 2016). This suggests that a contrasting seasonal moisture signal carried by δ^18^O of earlywood and latewood. In the subtropical area of southwestern China, early vegetation period (April, May) is characterized by sunny conditions with little rainfall and low humidity, while ample monsoon precipitation and high humidity occurs during summer (**Figure 1**).

The correlations coefficients between reconstructed source water δ^18^O and the weighted mean δ^18^O of precipitation in Kunming were higher in earlywood growth period (EWG) than that of latewood period (LWG) for both pine species (Supplementary Figure S3), which were consistent with An et al. (2012), and the correlation coefficients was higher in EWG of *P. armandii* (*r* = 0.35) than that of *P. kesiya* (*r* = 0.25). However, the correlations between reconstructed δ^18^O and weighted mean of precipitation were much lower than other studies (Xu C. et al., 2014), which could be due to the long distance between Kunming station and our study sites as well as short time period of the available data (1986–2003).

## Conclusion

We found a strong carry-over effect for δ^13^C in both secondary forest pine species *P. kesiya* and natural forest pine species *P. armandii*. In *P. kesiya*, there was no significant difference between earlywood and latewood for either δ^13^C or δ^18^O, however, both δ^13^C and δ^18^O in the earywood were significantly higher than those of latewood in *P. armandii*. Our results showed that *P. armandii* had slightly higher iWUE in than that of *P. kesiya*. Water use efficiency is increasing in both pine species, however, with a higher rate in *P. kesiya*. δ^13^C variations in earlywood and latewood differed among the studied species and represented a site-specific or species-specific climate signals. Due to the impact of resin collection on *P. kesiya*, care should be taken to interpret the correlations relationships between tree-ring δ^13^C and climate factors. In contrast to δ^13^C, δ^18^O variations showed strong coherence between the study species. δ^18^O_EW_ of both pine species was negatively correlated with May RH, whereas δ^18^O_LW_ of both species had a strong significant autumn precipitation signal and thus has the potential to reconstruct autumn precipitation in the study area. The contrasting climatic signals in δ^18^O_EW_ and δ^18^O_LW_ imply that separate analyses of earlywood and latewood are recommendable to derive clear climatic signals in δ^18^O series from conifer species growing in the mid-mountain zone of the summer monsoon region of southwestern China.

## Author Contributions

AB, AG, Z-XF, and P-LF designed the experiment. P-LF, JG, and AG performed the experiment. P-LF and Z-XF analyzed the data. All authors contributed to the writing of the manuscript.

## Conflict of Interest Statement

The authors declare that the research was conducted in the absence of any commercial or financial relationships that could be construed as a potential conflict of interest.
