# Corrigendum: Earlywood and Latewood Stable Carbon and Oxygen Isotope Variations in Two Pine Species in Southwestern China during the Recent Decades

**DOI:** 10.3389/fpls.2017.00923

**Published:** 2017-05-31

**Authors:** Pei-Li Fu, Jussi Grießinger, Aster Gebrekirstos, Ze-Xin Fan, Achim Bräuning

**Affiliations:** ^1^Key Laboratory of Tropical Forest Ecology, Xishuangbanna Tropical Botanical Garden, Chinese Academy of SciencesMenglun, China; ^2^Institute of Geography, University of Erlangen-NürnbergErlangen, Germany; ^3^World Agroforestry CentreNairobi, Kenya

**Keywords:** stable carbon isotope, stable oxygen isotope, intrinsic water use efficiency, subtropical pine species, Asian summer monsoon, intra-annual resolution

In the original article, there was a mistake in Figure 4 as published. The unit of the y-axis in Figures 4A,B should be μmol mol^−1^, not μmol s^−2^ s^−1^. The corrected Figure 4 appears below. The authors apologize for this error and state that this does not change the scientific conclusions of the article in any way.

**Figure 4:**
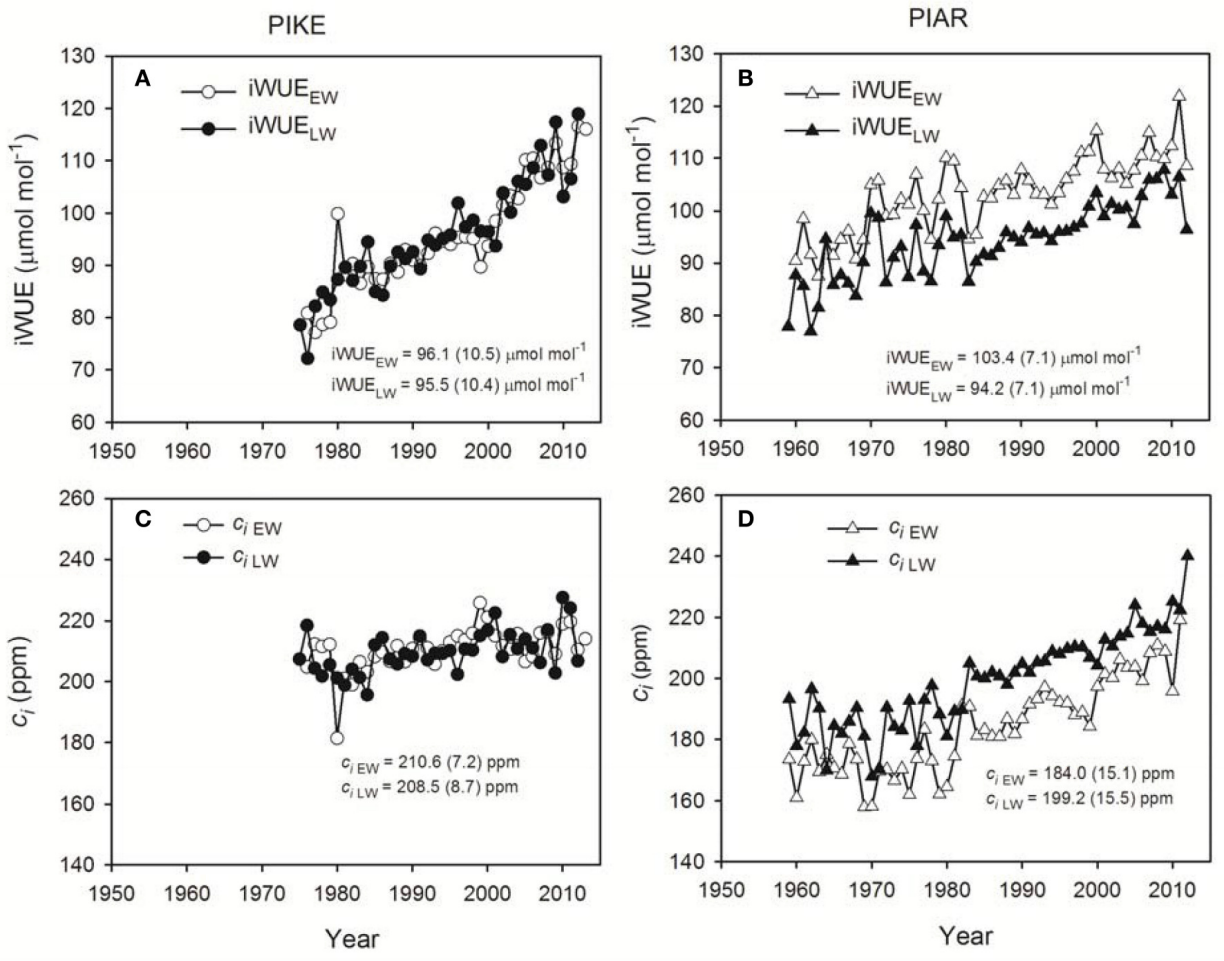
Intrinsic water use efficiency (iWUE) for earlywood (iWUE_EW_, open circle) and latewood of secondary forest pine *P. kesiya* (PIKE) (iWUE_LW_, closed circle) **(A)**, and for earlywood (iWUE_EW_, open triangle) and latewood of natural forest pine *P. armandii* (PIAR) (iWUE_LW_, closed triangle) **(B)**, intercellular CO_2_ concentration (c_*i*_) of earlywood (ci_EW_, open circle) and latewood of secondary forest pine *P. kesiya* (ci_LW_, closed circle) **(C)**, and for earlywood (ci_EW_, open triangle) and latewood of natural forest pine Pinus armandii (ci_LW_, closed triangle) **(D)**. Mean values and standard deviations of iWUE and c_*i*_ series are indicated inside the panels.

## Conflict of interest statement

The authors declare that the research was conducted in the absence of any commercial or financial relationships that could be construed as a potential conflict of interest.

