# Supplementary material for: Earlywood and Latewood Stable Carbon and Oxygen Isotope Variations in Two Pine Species in Southwestern China during the Recent Decades
Source: Front Plant Sci. 2017 Jan 10;7:2050. doi: 10.3389/fpls.2016.02050 (PMC5223062; doi:10.3389/fpls.2016.02050)
Supplement: Supplementary file 1 [file Data_Sheet_1.docx]

**Supplementary Material**

**Table S1** Pearson’s correlation coefficients of monthly mean temperature (TEM), preciptation (PRE) and relative humidity (RH) between Jingdong County and Ailaoshan Station for Subtropical Forest Ecosystem Studies (ALS) (1982-2012). Bold values indicate correlations significant at *P* < 0.05.

| **Month** | **TEM** | **PRE** | **RH** |
| --- | --- | --- | --- |
| **Jan** | **0.75** | **0.84** | **0.58** |
| **Feb** | **0.91** | **0.82** | **0.71** |
| **Mar** | **0.88** | **0.88** | **0.47** |
| **Apr** | **0.94** | **0.89** | **0.57** |
| **May** | **0.95** | **0.91** | **0.73** |
| **Jun** | **0.91** | **0.80** | **0.39** |
| **Jul** | **0.89** | **0.62** | 0.25 |
| **Aug** | **0.88** | **0.59** | 0.30 |
| **Sep** | **0.91** | **0.73** | 0.37 |
| **Oct** | **0.65** | **0.81** | 0.25 |
| **Nov** | **0.78** | **0.94** | 0.19 |
| **Dec** | **0.83** | **0.70** | **0.40** |
| **Annual** | **0.80** | **0.73** | **0.38** |
| **Mean R** | 0.81 | 0.79 | 0.43 |


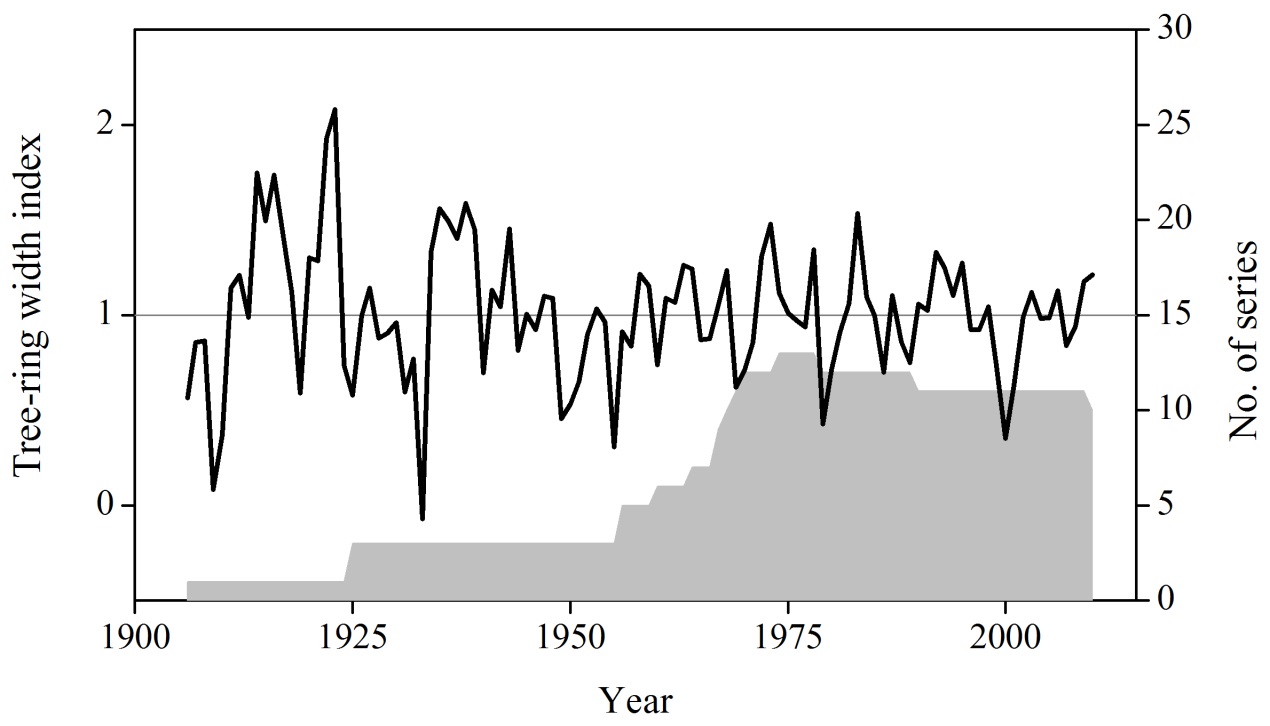


**Figure S1** The standard tree-ring width chronologies and number of series of *P. armandii* in the Ailao Mountains. Tree-ring measurements were detrended with a cubic spline function with a 50% frequency-response cut-off equal to 67% the series length. The resulting detrended series were averaged by computing the bi-weight robust mean.


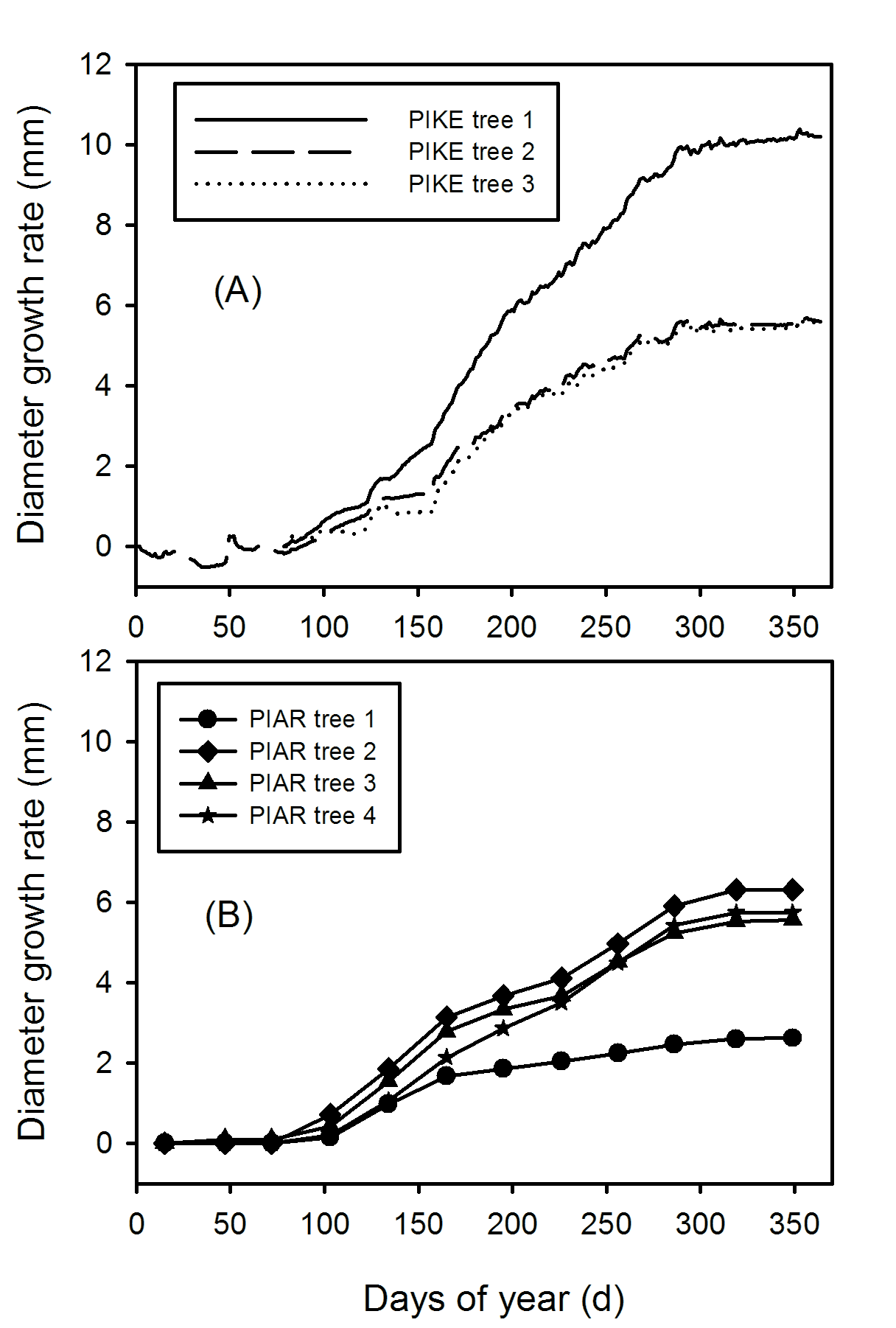


**Figure S2** The intra-annual diameter growth rate of secondary forest pine *P. kesiya* (A, monitored with high resolution dendrometer) and natural forest pine *P. armandii* (B, measured with band dendrometer).


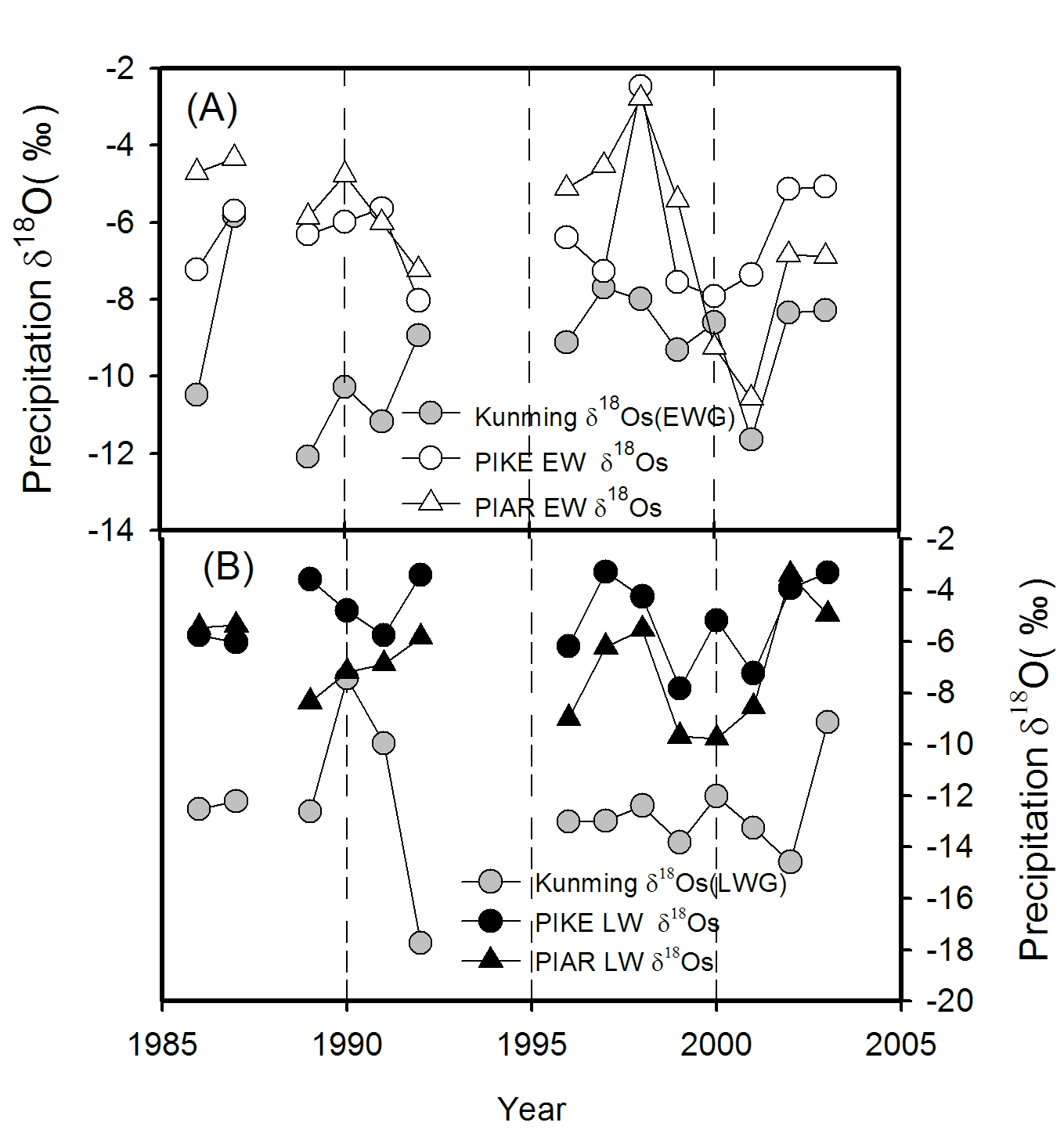


**Figure S3** The reconstruction of source water δ^18^O_S_ in the earlywood (April-July) and latewood (August to October) growth period of *P. kesiya* (PIKE) and *P. armandii* (PIAR) and the weighted mean δ^18^O of precipitation at the nearest Global Network of Isotopes in Precipitation (GNIP) station, in Kunming (World Metrological Organization station #5677800; 25°02N′, 102°43′ E, at 1895 m a.s.l.) from 1986 to 2003. We used the fraction model of Waterhouse et al. (2002) and follow the method of An et al. (2012) to reconstruct the earlywood and latewood growth period source water δ^18^O.

δ^18^O_cell_ = (1-*f*_0_) [δ^18^O_S_+ (ε_e_+ε_k_) (1-*h*) + ε_0_] + *f*_0_ (δ^18^O_S_+ε_0_)] (1)

δ^18^O_S_ =δ^18^O_cell_ -ε_0_ - (1- *f*_0_) (ε_e_+ε_k_) (1-*h*) (2)

*f*_0_ is a damping factor, and we use the value 0.40 as Gessler et al. (2009) found that there is 40% exchange between organic oxygen and xylem water oxygen during the cellulose synthesis. ε_e_ is the equilibrium fractionation factor, we use the value 9‰ following Allison et al. (1985). For ε_k_ the kinetic fractionation factor, and ε_0_ the biological fractionation factor, we used the values of 28 ‰ and 27 ‰, respectively, as suggested by (Anderson et al., 2002). *h* is relative humidity, we used the average values *h* of earlywood and latewood growth period, respectively. We used the *h* from Ailaoshan Station for Subtropical Forest Ecosystem Studies (ASSFES) during 1986-2003.

References:

Allison, G.B., Gat, J.R., and Leaney, F.W.J. (1985). The relationship between deuterium and oxygen-18 delta values in leaf water. *Chem. Geol.* 58, 145–156. doi:10.1016/0168-9622(85)90035-1.

An, W., Liu, X., Leavitt, S.W., Ren, J., Sun, W., Wang, W., et al. (2012). Specific climatic signals recorded in earlywood and latewood δ18O of tree rings in southwestern China. Tellus B 64, 18703. doi: 10.3402/tellusb.v64i0.18703.

Anderson, W.T., Bernasconi, S.M., McKenzie, J.A., Saurer, M., and Schweingruber, F. (2002). Model evaluation for reconstructing the oxygen isotopic composition in precipitation from tree ring cellulose over the last century. *Chem. Geol.* 182, 122–137. doi: 10.1016/S0009-2541(01)00285-6.

Gessler, A., Brandes, E., Buchmann, N., Helle, G., Rennenberg, H., and Barnard, R.L. (2009). Tracing carbon and oxygen isotopes signals from newly assimilated sugars in the leaves to tree-ring archive. *Plant Cell Environ.* 32, 780–795. doi: 10.1111/j.1365-3040.2009.01957.x.

Waterhouse, J. S., Switsur, V. R., Barker, A. C., Carter, A.H.C., and Robertson, I. 2002. Oxygen and hydrogen isotope ratios in tree rings: how well do models predict observed values? *Earth Planet Sci. Lett.* 201, 421–430. doi: 10.1016/S0012-821X(02)00724-0.
